# Hypoxia confers ferroptosis resistance in glioma cells via HIF-1α/SREBP1-mediated regulation of lipid metabolism

**DOI:** 10.1016/j.jlr.2026.101139

**Published:** 2026-09-01

**Authors:** Zhongjun Shen, Yao Zhao, Mingbo Jia, Minghao Shi, Yang Yang, Ying Sun, Yao Li, Xiaoyi Liu, Zhuoyue Shan, Liyan Zhao

**Affiliations:** 1 Department of Blood Transfusion, Second Hospital of Jilin University, Changchun, Jilin, China; 2 Department of Neurosurgery, First Hospital of Jilin University, Changchun, Jilin, China

**Keywords:** glioma, hypoxia-inducible factor-1α, ferroptosis, FASN, SCD1, SREBP1

## Abstract

Glioma represents one of the most aggressive tumors in the central nervous system, with clinical management facing significant challenges including high recurrence rates and therapeutic resistance. Ferroptosis, an iron-dependent form of cell death, holds potential for glioma treatment, yet tumor cells frequently develop evasion mechanisms. This study elucidates the molecular mechanisms by which hypoxic microenvironment confers ferroptosis resistance in glioma cells, focusing on the pivotal role of the hypoxia-inducible factor 1α (HIF-1α)/SREBP1 signaling axis and its downstream effectors FASN and stearoyl-CoA desaturase-1 (SCD1). Our experimental results demonstrate that hypoxic conditions significantly upregulate HIF-1α expression and confer resistance to RSL3-induced ferroptosis. Mechanistic studies reveal that HIF-1α promotes SREBP1 activation, which subsequently upregulates FASN and SCD1 expression to suppress lipid peroxidation.Furthermore, the HIF-1α-specific inhibitor PX-478 effectively reverses hypoxia-induced ferroptosis resistance and significantly enhances tumor cell sensitivity to ferroptosis inducers. *In vivo* experiments confirm the potent antitumor effects of PX-478 combined with RSL3. This study systematically elucidates the role of the HIF-1α-SREBP1-FASN/SCD1 signaling axis in ferroptosis regulation in glioma, providing important theoretical foundations and experimental support for developing HIF-1α-targeted ferroptosis therapies.

## Graphical abstract

**Figure:**
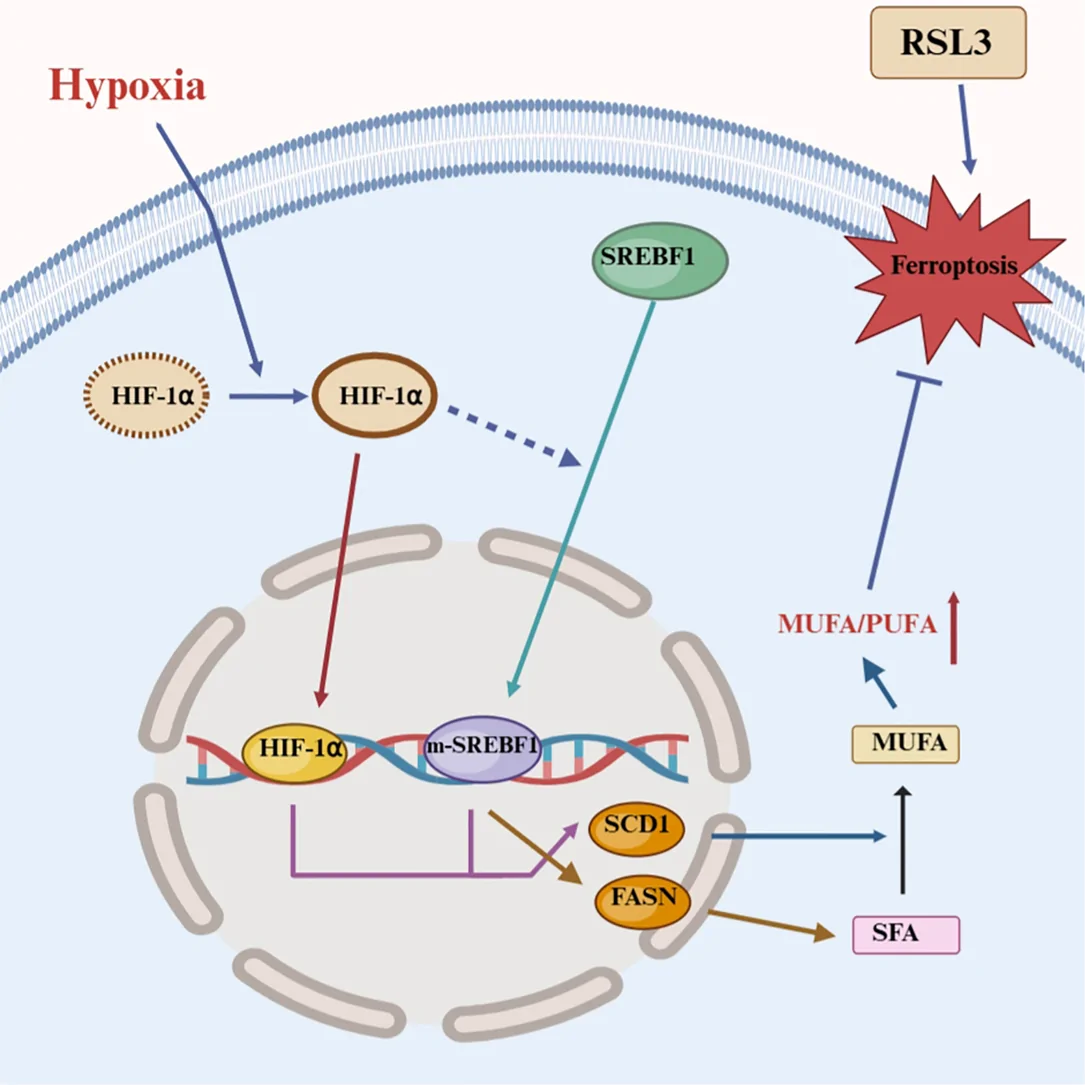

Gliomas represent one of the most aggressive and malignant tumors of the central nervous system, characterized by rapid progression, therapeutic resistance, and high recurrence rates (1, 2). Despite significant advancements in surgical resection, radiotherapy, and chemotherapy, patient survival remains unsatisfactory due to tumor drug resistance and posttreatment recurrence (3).

Hypoxia within the tumor microenvironment serves as a critical adaptive mechanism for glioma cell survival under adverse conditions. Beyond influencing cellular energy metabolism, hypoxia promotes tumor progression and drug resistance by regulating diverse biological processes (4, 5). Hypoxia-inducible factors (HIFs), particularly HIF-1α, function as master regulators that activate downstream genes to modulate tumor cell metabolism, proliferation, invasion, and antiapoptotic capacity (6, 7, 8, 9). HIF-1α and its target genes correlate with pathological grading, tumor progression, and therapeutic resistance in brain tumors, with their expression associated with poor patient prognosis (4, 5).

Metabolic reprogramming driven by hypoxia constitutes a key driver of glioma survival and progression (10). As a crucial component of tumor metabolic remodeling, lipid metabolism significantly influences membrane stability and energy homeostasis. SREBP1 serves as the principal transcriptional regulator of lipid metabolism, maintaining lipid homeostasis through control of fatty acid and cholesterol synthesis pathways (11, 12, 13). Under hypoxic conditions, HIF-1α upregulates SREBP1 expression to promote lipid synthesis and membrane biogenesis, thereby enhancing tumor cell survival (14, 15).

FASN and stearoyl-CoA desaturase-1 (SCD1) emerge as key enzymes in lipid metabolism under SREBP1 regulation. FASN catalyzes de novo fatty acid synthesis to provide lipid precursors for tumor growth (16), while SCD1 converts saturated fatty acids (SFAs) to MUFAs, modulating membrane composition and functionality (17). Both enzymes demonstrate elevated expression across multiple cancers and correlate with tumor malignancy and poor prognosis (18).

Ferroptosis, an iron-dependent programmed cell death characterized by aberrant lipid peroxide accumulation and membrane oxidative damage (19), exhibits intimate connections with lipid metabolism. Upregulation of FASN and SCD1 not only maintains membrane stability but also confers ferroptosis resistance (20). SCD1 reduces lipid peroxidation susceptibility by increasing MUFA content in cellular membranes (21). Notably, the incorporation of MUFAs into membrane phospholipids requires their prior activation by acyl-CoA synthetase long-chain family member 3 (ACSL3), and genetic ablation of ACSL3 has been shown to abolish the ferroptosis-protective effect of MUFAs (22). Meanwhile, FASN supplies adequate fatty acids to support membrane integrity against ferroptotic damage (23).

Although existing studies suggest HIF-1α-mediated ferroptosis suppression in glioma cells (24), the mechanistic interplay between HIF-1α-regulated lipid metabolism and ferroptosis resistance remains poorly understood. Elucidating how the HIF-1α/SREBP1 axis modulates FASN and SCD1 to promote lipid metabolic adaptation and ferroptosis resistance under hypoxia will provide critical insights into glioma survival strategies and facilitate development of novel therapeutic targets.

## Materials and methods

### Patients and tissue samples

Tumor specimens were collected from 10 glioma patients who underwent surgical resection at the Department of Neuro-Oncology Surgery, The First Hospital of Jilin University. All patients had not received prior radiotherapy or chemotherapy, and the diagnosis of glioma was confirmed by postoperative histopathological examination. This study was conducted in accordance with the Declaration of Helsinki and was approved by the Ethics Committee of The First Hospital of Jilin University. Written informed consent was obtained from all participants.

### Immunohistochemical staining

Paraffin-embedded tissue sections were deparaffinized and rehydrated, followed by antigen retrieval using heat-mediated methods. Endogenous peroxidase activity was blocked, and the sections were incubated with primary antibodies at 4°C overnight in a humidified chamber. The next day, HRP-conjugated goat anti-mouse/rabbit IgG secondary antibodies were applied and incubated for 30 min at room temperature. Diaminobenzidine was used for chromogenic detection, followed by counterstaining with hematoxylin. Finally, the sections were mounted and imaged under a microscope. Five random fields per section were captured for semiquantitative analysis using ImageJ software.

### Cell culture

Human glioma cell lines (U251MG, LN229) and 293T cells were cultured in DMEM supplemented with 5% FBS and 1% penicillin-streptomycin at 37°C in a humidified 5% CO_2_ incubator (normoxic conditions). To mimic the hypoxic tumor microenvironment, U251MG and LN229 cells were cultured in a tri-gas incubator (37°C, 5% CO_2_, 1% O_2_, and 94% N_2_) for 24 h prior to and during treatments for subsequent hypoxia experiments. The 293T cell line was provided by the Neuro-Oncology Surgery Laboratory, while U251MG and LN229 cells were purchased from iCell Bioscience Inc. All cell lines were authenticated by short tandem repeat profiling and routinely tested for mycoplasma contamination.

### Reagents and antibodies

Deferoxamine (DFO) was purchased from Sigma-Aldrich, dissolved in PBS to 100 mM stock solution and diluted with culture medium to 10 μM for treatment. RSL3 was purchased from MedChemExpress, dissolved in DMSO to 10 mM stock solution. A939572 was purchased from TargetMol, dissolved in DMSO to 10 mM stock solution. PX-478 was purchased from Selleck, dissolved in PBS to 10 mM stock solution. Anti-HIF-1α was purchased from Cell Signaling Technology. Anti-SCD1, anti-SREBP1, and anti-FASN were purchased from Proteintech, Abcam and CST, respectively. All primary antibodies were diluted 1:1000 for Western blot and 1:200 for immunohistochemistry. β-Tubulin was purchased from OriGene and diluted 1:5000 for Western blot. Goat anti-rabbit IgG and goat anti-mouse IgG were purchased from Yeasen and diluted 1:5000 for Western blot. For oleic acid (OA) treatment, OA (Sigma-Aldrich) was conjugated with fatty acid-free BSA at a molar ratio of 6:1 according to the manufacturer's protocol. The OA-BSA conjugate was then added to cell culture medium at a final concentration of 10 μM. Cells were treated with OA simultaneously with RSL3 for 24 h under hypoxic conditions.

### LC-MS/MS method for lipid analysis

Reverse phase chromatography was selected for LC separation using Cortecs C18 column (2.1∗100 mm, 2.7um, Waters). Mobile phase A was made by mixing 400 ml of HPLC-grade water containing 0.77 g of ammonium acetate with 600 ml of HPLC-grade acetonitrile. Mobile phase B contained 10% ACN and 90% IPA (v/v). Gradient elution was performed at a flow rate of 0.25 ml/min, and the column oven temperature was set to 40 °C. The gradient was as follow: 0 min, 30%B; 3.0 min, 30% B; 4.5 min, 33% B; 7.0 min, 45% B; 8.0 min, 52% B; 11.0 min, 58% B; 14.0 min, 66% B; 17.0 min, 70% B; 21.0 min, 75% B; 23.0 min, 98% B; 30.0 min, 98% B; 30.5 min, 30% B; 35.0 min, 30% B.

Data were acquired using Orbitrap Exploris 240 mass spectrometer (Thermo Fisher Scientific, CA) coupled with UHPLC system Vanquish (Thermo Fisher Scientific, CA). The detailed mass spectrometer parameters were as follows: spray voltage, 3.2 kV for positive and 2.8 kV for negative; capillary temperature, 320°C; sheath gas flow rate (arb),35; aux gas flow rate (arb),10; mass range (m/z), 240–2000 for positive and 200–2000 for negative. Full MS resolution, 60,000; MS/MS resolution, 15,000; NCE, 15/30/45; duty cycle, 1s.

### Lipid identification using LipidSearch

Lipids were identified and quantified using LipidSearch 4.2 (Thermo Fisher Scientific).Lipid Search v4.2 contains 18 lipid species and more than 1,500,000 fragment ions in the database. It allows lipid identification based on MS/MS match. Mass tolerance for precursor and fragment was set to 8 ppm and 10 ppm respectively. The displayed m-score threshold was selected as 5 and grades A, B, C, and D were all used for ID quality filter. All lipid classes in database including 71 subspecies were chosen for identification. Adducts of +H, +NH4 were applied for positive mode search and –H, +CH_3_COO were selected for negative mode since ammonium acetate was used in mobile phases. Only the lipids with chromatographic area >5E6 were considered as confident identification. 0.25 min retention time shift was allowed for quantitation.

### Western blot analysis

Cells were lysed in RIPA buffer supplemented with protease and phosphatase inhibitors, followed by sonication and incubation on ice for 30 min. After centrifugation at 14,000 *g* for 30 min at 4°C, supernatants were collected and protein concentrations were determined using the BCA assay. Protein samples were mixed with 5× SDS loading buffer, denatured at 95°C for 5 min, and stored at −80°C. Proteins were separated by SDS-PAGE and transferred to PVDF membranes. After blocking, membranes were incubated with primary antibodies overnight at 4°C, followed by incubation with HRP-conjugated secondary antibodies for 1 h at room temperature. Protein bands were visualized using ECL substrate and quantified using ImageJ software.

### Quantitative real-time PCR (qPCR)

Total RNA was extracted using TRIzol reagent and reverse transcribed into cDNA using Hifair™ III first Strand cDNA Synthesis SuperMix according to the manufacturer's protocol. Quantitative real-time PCR (qPCR) was performed using Hieff™ qPCR SYBR Green Master Mix. Relative gene expression was calculated using the 2^-ΔΔCt^ method. Primer sequences are provided in Supplementary Information.

### siRNA transfection

Gene silencing was performed using target-specific siRNAs. siRNA-transfection reagent complexes were prepared in serum-free medium and added to adherent cell cultures. Transfected cells were incubated for 24–72 h at 37°C. Knockdown efficiency was validated by qPCR and Western blot using β-actin as internal control. siRNA sequences are listed in Supplementary Information.

### Plasmid transfection and lentiviral infection

SREBF1 shRNA plasmids were designed and synthesized by Transheep Bio. For lentiviral production, 293T cells were cotransfected with packaging plasmids (psPAX2 and pMD2.G). Viral supernatants were collected to infect U251MG and LN229 cells in the presence of Polybrene (8 μg/ml). Stable transfectants were selected using appropriate antibiotics. Target sequences are provided in Supplementary Information.

### Cell viability assay

Cell viability was assessed using CCK-8 assay. Transfected cells were seeded in 96-well plates and incubated with CCK-8 reagent for 2 h at 37°C. Absorbance was measured at 450 nm. Cell viability (%) was calculated as: (Aexperiment - Ablank)/(Acontrol - Ablank) × 100%.

### Lipid peroxidation assay

Malondialdehyde (MDA) levels were measured using a commercial kit according to the manufacturer's instructions. Absorbance was measured at 532 nm and MDA concentrations were determined using a standard curve.

### Lactate dehydrogenase (LDH) release assay

Cytotoxicity was evaluated by measuring lactate dehydrogenase (LDH) activity in culture supernatants using a colorimetric assay. Cell death rate (%) was calculated as: (Asample - Acontrol)/(Amax - Acontrol) × 100%, where Amax represents LDH activity in lysed control cells.

### In vivo experiments

Subcutaneous xenograft models were established by inoculating 2 × 10^6^ U251MG cells into the right flanks of nude mice. When tumor volumes reached ∼100 mm^3^, mice were randomly divided into four treatment groups (n = 6/group): (1) saline control, (2) PX-478 (15 mg/kg), (3) RSL3 (10 mg/kg), and (4) PX-478+RSL3 combination. Drugs were administered via intraperitoneal injection every 3 days for 16 days. The experiment was terminated when tumor volumes reached 1,000 mm^3^. All animal experimental procedures were approved by The Animal Experimental Ethical Committee of the School of Public Health, Jilin University. All methods were carried out in accordance with relevant guidelines and regulations. All efforts were made to minimize animal suffering.

### Statistical analysis

Data were analyzed using SPSS 20.0 and GraphPad Prism 8.0. Comparisons between two groups were performed using unpaired Student's *t* test. Multiple group comparisons were analyzed by one-way ANOVA followed by SNK-q post hoc test. Data are presented as mean ± SD from three independent experiments. *P* < 0.05 was considered statistically significant.

## Results

### Hypoxia confers ferroptosis resistance in Glioma cells

In order to investigate the effect of hypoxia on ferroptosis in glioma cells, U251MG and LN229 cell lines were cultured under normoxia (21% O_2_) and hypoxic (1% O_2_) conditions, respectively, and treated with ferroptosis inducer RSL3. CCK-8 experiments showed that RSL3 inhibited cell viability in a concentration-dependent manner under normoxic conditions, with an IC50 of approximately 5 μM (Fig. 1A, B). Under hypoxic conditions, the cytotoxicity of the same concentration of RSL3 was significantly reduced and the cell survival rate was significantly increased (Fig. 1C, D). In addition, the ferroptosis inhibitor DFO (10 μM) can further enhance cell survival under hypoxia, suggesting that hypoxia may promote cell survival by inhibiting ferroptosis.

**Fig. 1:**
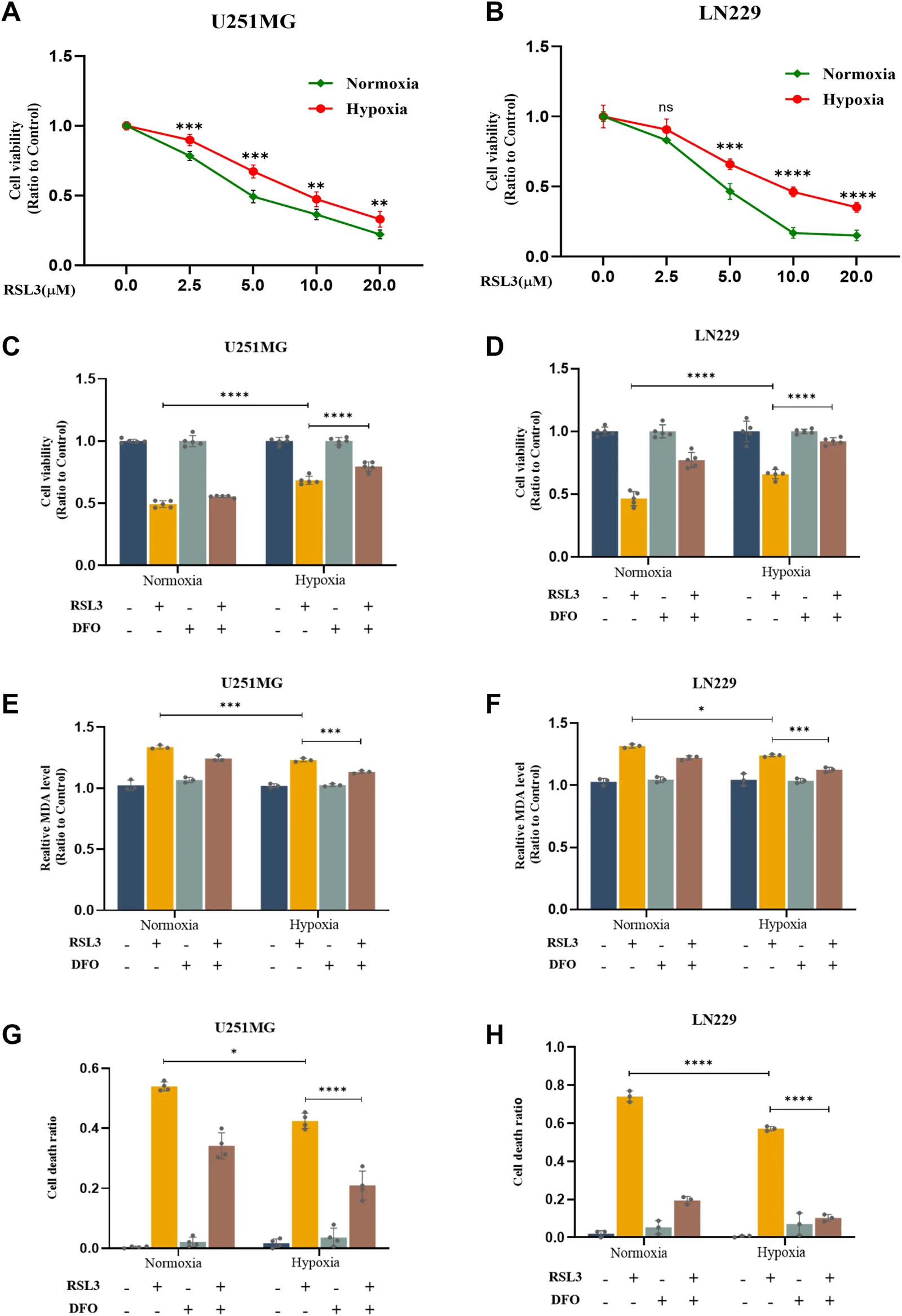
Hypoxia inhibits ferroptosis in glioma cells. A and B: Cells were treated with different concentrations of RSL3 (0, 2.5, 5, 10, and 20 μM) for 24 h under normoxia and hypoxic conditions, and cell viability was detected by CCK-8. C and D: Cells were treated with RSL3 (5 μM), DFO (10 μM), or a combination of them under normoxia and hypoxic conditions, and cell viability was detected by CCK-8. E and F: Cells were treated with RSL3, DFO, or their combination under normoxic and hypoxic conditions, and their peroxidation degree was detected by lipid peroxide (MDA). G and H: Cells were treated with RSL3, DFO, or their combination under normoxia and hypoxic conditions, and the degree of cell death was detected by LDH.∗*P* < 0.05,∗∗*P* < 0.01,∗∗∗*P* < 0.001,∗∗∗∗*P* < 0.0001. DFO, deferoxamine; LDH, lactate dehydrogenase.

To further verify the effect of hypoxia on ferroptosis, we measured lipid peroxidation levels (MDA) and cytotoxicity (LDH release). The results showed that hypoxia significantly reduced RSL3-induced MDA accumulation (Fig. 1E, F) and LDH release (Fig. 1G, H) compared with normoxic group, indicating that hypoxia effectively inhibited lipid peroxidation and cell membrane damage associated with ferroptosis. Together, these results suggest that hypoxia enhances glioma cell survival by inhibiting ferroptosis, laying the foundation for subsequent mechanistic studies.

### Hypoxia alters the lipid metabolism profile of glioma cells and promotes the accumulation of MUFAs

In order to further explore the basis of lipid metabolism in the inhibition of ferroptosis by hypoxia, we performed non-targeted lipidomics analysis of U251MG under normoxia and hypoxic conditions. As shown in Fig. 2A, hypoxia causes significant changes in fatty acid composition in a variety of phospholipid groups, including cardiolipin (CL), phosphatidylglycerol (PG), phosphatidylcholine (PC), phosphatidylethanolamine (PE), phosphatidylserine (PS), and phosphatidylinositol (PI).

**Fig. 2:**
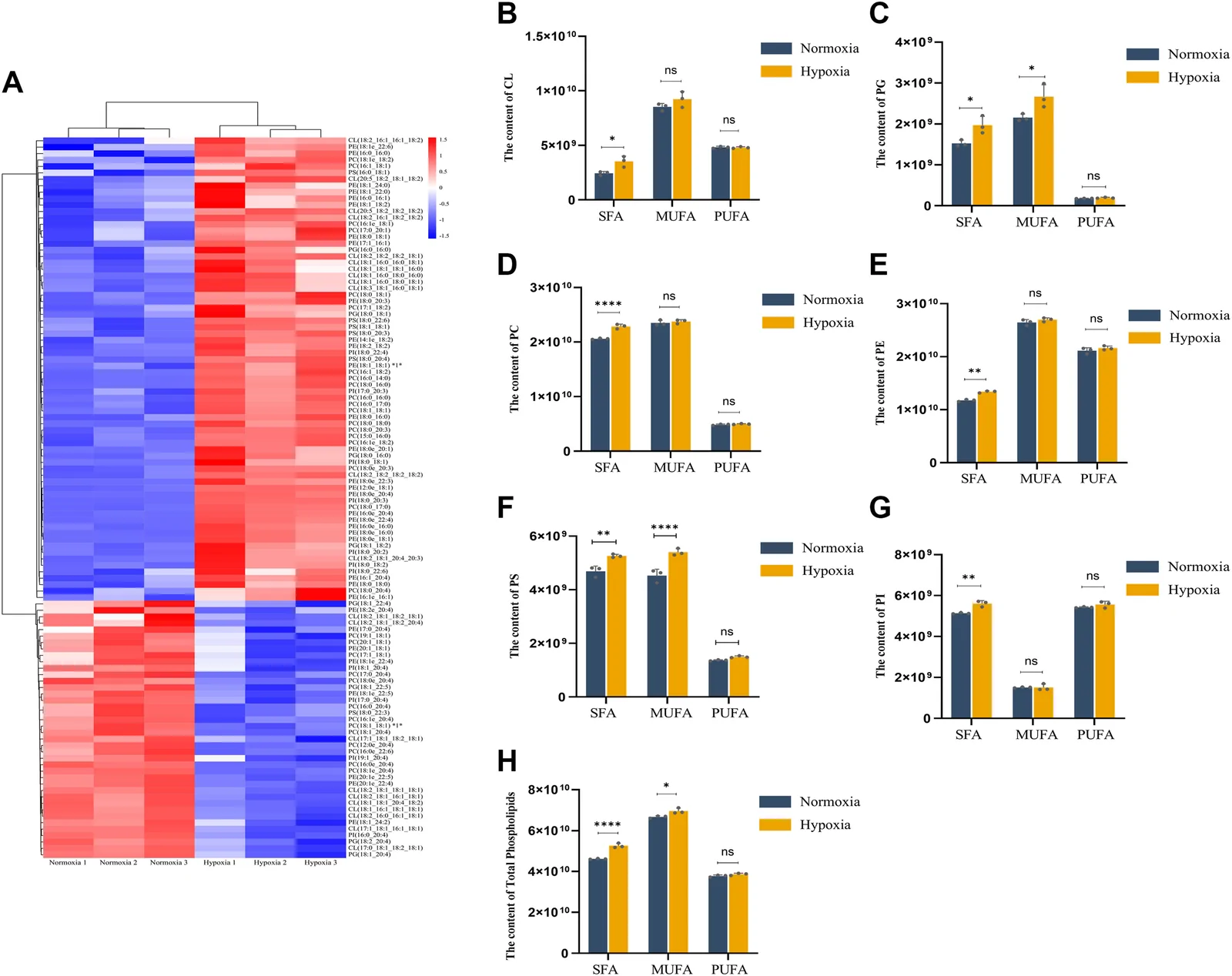
Hypoxia alters the lipid metabolism profile of glioma cells and promotes the accumulation of monounsaturated fatty acids. A: The cells were cultured under normoxia and hypoxia conditions for 24 h, and the lipids were presented for non-targeted lipidomics detection and analysis, and the fatty acids with differences in content between groups were analyzed and heat mapped. B–G: Hypoxia-treated glioma cells were treated with phospholipids (CL), phosphatidylglycerol (PG), phosphatidylcholine (PC), phosphatidylethanolamine (PE), phosphatidylserine (PS) and phosphatidylinositol (PI) for quantitative analysis of saturated fatty acids (SFAs), monounsaturated fatty acids (MUFAs) and polyunsaturated fatty acids (PUFAs). H: All saturated fatty acids (SFAs), monounsaturated fatty acids (MUFAs), and polyunsaturated fatty acids (PUFAs) in hypoxia-treated glioma cells were quantitatively analyzed.∗*P* < 0.05,∗∗*P* < 0.01,∗∗∗*P* < 0.001,∗∗∗∗*P* < 0.0001.

We observed that SFAs were generally elevated in a variety of phospholipids (Fig. 2B–G), while MUFAs were significantly increased in PG and PS (Fig. 2C, F), but overall, the proportion of total SFA and MUFA was increased in hypoxic cells (Fig. 2H), suggesting that SFA and MUFAs play an important role in glioma cells under hypoxic conditions. FASN catalyzes de novo synthesis of fatty acids, providing tumor cells with lipid precursors needed for growth. SCD1 regulates the lipid composition and function of cell membranes by converting SFA to MUFA, and given that MUFA can incorporate membrane phospholipids and reduce lipid peroxidation sensitivity, we speculate that MUFA accumulation may play a key role in hypoxia-induced ferroptosis resistance.

To determine whether the hypoxia-induced increase in MUFA levels, although modest in magnitude, is functionally sufficient to confer ferroptosis resistance, we performed ACSL3 knockdown experiments. ACSL3 catalyzes the conversion of free MUFAs to MUFA-CoA, an essential step for their incorporation into membrane phospholipids. U251MG and LN229 cells were transfected with ACSL3 siRNA for 24 h (supplemental S1A, B) and then treated with RSL3 under normoxic or hypoxic conditions. Knockdown of ACSL3 significantly sensitized both cell lines to RSL3-induced ferroptosis under both normoxia and hypoxia, as evidenced by decreased cell viability, increased lipid peroxidation (MDA), and elevated LDH release (supplemental S1C–H). Notably, under hypoxic conditions, ACSL3 depletion largely reversed the ferroptosis resistance conferred by hypoxia, with cell death and lipid peroxidation levels approaching those observed in normoxic cells treated with RSL3 alone. These findings demonstrate that the hypoxia-elevated MUFA pool, once incorporated into membrane phospholipids via ACSL3, is functionally significant and sufficient to mediate a meaningful degree of ferroptosis resistance, despite the relatively modest increase detected by bulk lipidomics.

### De novo lipid synthesis key enzymes FASN and SCD1 were upregulated and mediated ferroptosis resistance under hypoxia

Based on the previous lipidomics results, we speculate that hypoxia may alter fatty acid composition by promoting de novo lipid synthesis. qPCR and Western blot results showed that the expression of FASN and SCD1 was significantly upregulated in both U251MG and LN229 cells under hypoxic conditions (Fig. 3A, B).

**Fig. 3:**
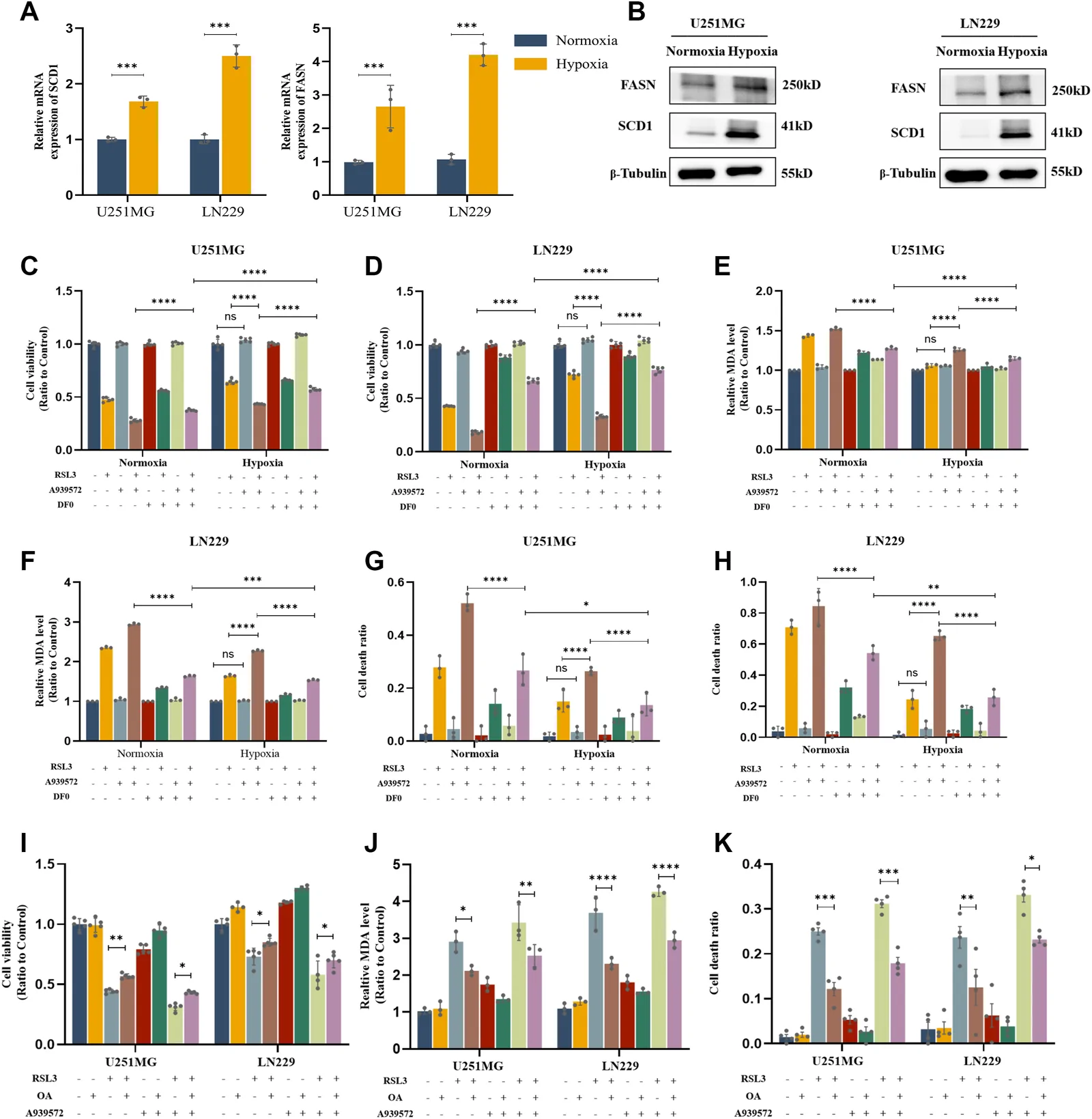
De novo lipid synthesis key enzymes FASN and SCD1 were upregulated and mediated ferroptosis resistance under hypoxia. A: Cells were cultured under normoxic and hypoxic conditions for 24 h, and the mRNA levels of SCD1 and FASN were detected by quantitative real-time PCR (qPCR). B: Cells were cultured under normoxic and hypoxic conditions for 24 h, and the protein levels of SCD1 and FASN were detected by Western blot. Protein quantification is shown in Supplementary file Figure 2A, B. C and D: Cells were cultured under normoxic and hypoxic conditions, and cells were treated with RSL3, A939572 (10 μM), DFO, or a combination of them for 24 h, and cell viability was detected by CCK-8. E and F: The cells were cultured under normoxic and hypoxic conditions, treated with RSL3, A939572, DFO or a combination of them for 24 h, and their peroxidation was detected by lipid peroxide (MDA). G and H: The cells were cultured under normoxia and hypoxia conditions, and the cells were treated with RSL3, A939572, DFO or a combination of them for 24 h, and the degree of cell death was detected by LDH. I–K: The cells were cultured under hypoxic conditions, and the cells were treated with RSL3, A939572, OA (10 μM), or their combination for 24 h, and cell viability was detected by CCK-8, the degree of peroxidation was detected by lipid peroxide (MDA), and the degree of cell death was detected by LDH.∗*P* < 0.05,∗∗*P* < 0.01,∗∗∗*P* < 0.001,∗∗∗∗*P* < 0.0001. SCD1, stearoyl-CoA desaturase-1; DFO, deferoxamine.

To verify SCD1 function, we treated cells with its inhibitor A939572. The results showed that inhibition of SCD1 significantly enhanced RSL3-induced cellular ferroptosis (Fig. 3C, D), MDA accumulation (Fig. 3E, F), and LDH release (Fig. 3G, H), and this effect could be partially reversed by the ferroptosis inhibitor DFO. In addition, exogenous supplementation with MUFA OA (10 μM) significantly alleviated the ferroptosis-inducing effect of RSL3 (Fig. 3I–K), indicating that SCD1 inhibits ferroptosis by catalyzing the production of MUFAs. Since FASN provides a catalytic substrate for SCD1, the FASN-SCD metabolic axis plays a central role in hypoxia-induced ferroptosis resistance.

### SREBP1 is essential for hypoxia-induced upregulation of SCD1 and FASN and mediates ferroptosis resistance via SCD1

To elucidate the role of SREBP1 in regulating SCD1 and FASN expression under hypoxic conditions, we first examined the effect of SREBF1 knockdown on the protein levels of these lipid-metabolizing enzymes. U251MG and LN229 cells were transfected with two independent shRNAs targeting SREBF1 (shSREBF1-1# and shSREBF1-2#) (supplemental S2C, D) and cultured under normoxic or hypoxic conditions for 24 h. Western blot analysis confirmed that hypoxia significantly induced the expression of mature SREBP1 (m-SREBP1), as well as its downstream targets SCD1 and FASN, in control cells (shNC). Importantly, knockdown of SREBP1 markedly attenuated the hypoxia-induced upregulation of both SCD1 and FASN proteins in both glioma cell lines (Fig. 4A, B). These results indicate that SREBP1 is required for the hypoxia-induced increase in SCD1 and FASN protein expression.

**Fig. 4:**
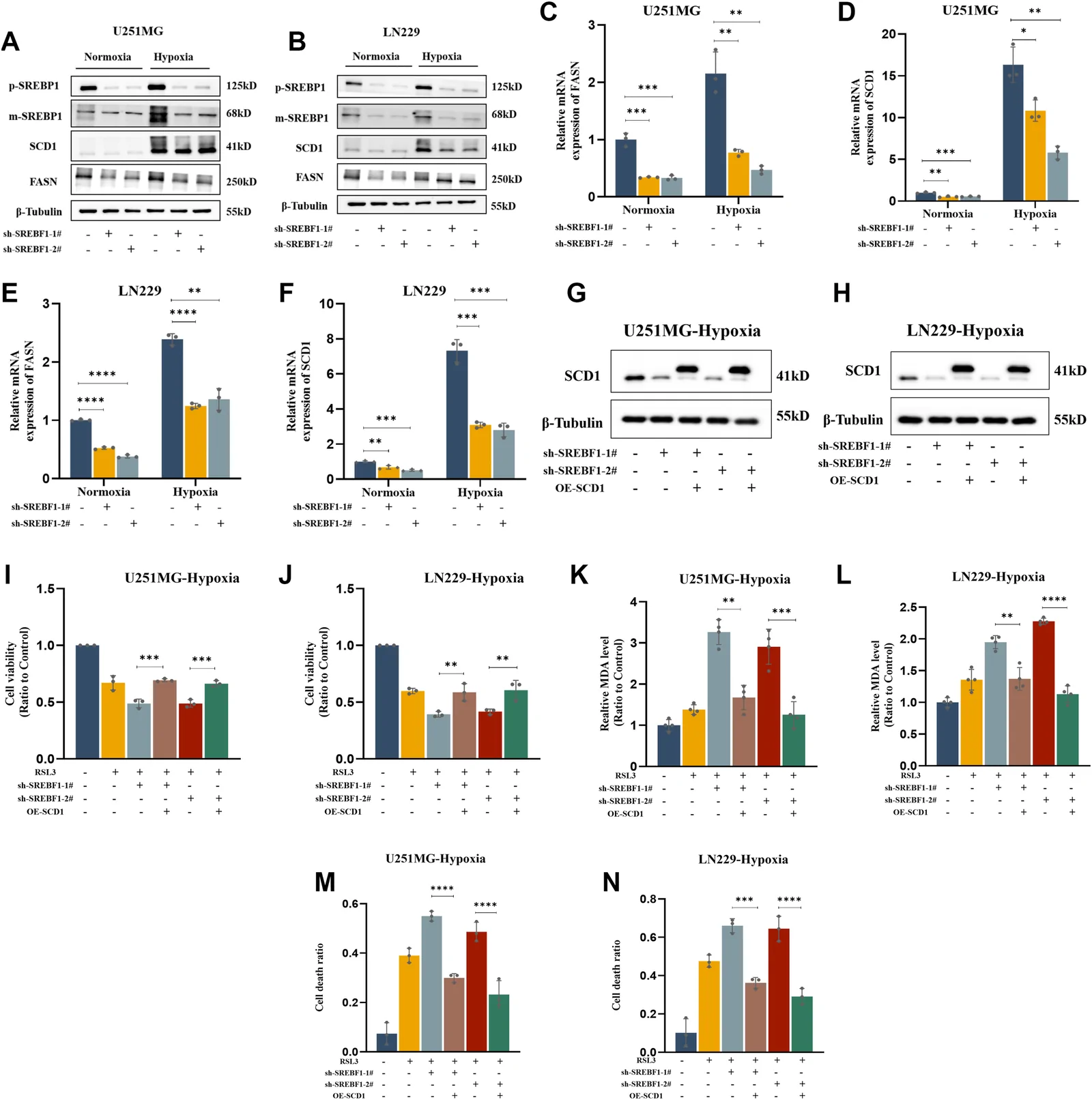
SREBP1 is essential for hypoxia-induced upregulation of SCD1 and FASN and mediates ferroptosis resistance via SCD1. A and B: Western blot analysis of m-SREBP1, SCD1, and FASN in U251MG (A) and LN229 (B) cells transfected with shNC or shSREBF1 (#1, #2) and cultured under normoxia (N) or hypoxia (H) for 24 h. Protein quantitative analysis is shown in Supplementary file Figure 2E–H. C–F: qPCR analysis of SCD1 (C, E) and FASN (D, F) mRNA levels in U251MG (C–D) and LN229 (E–F) cells treated as in (A–B). G and H: Western blot analysis of SCD1 in U251MG (G) and LN229 (H) cells transfected with shNC, shSREBF1, or shSREBF1+OE SCD1 under hypoxia. (I–N) CCK-8 (I–J), MDA (K–L), and LDH (M–N) assays in U251MG (I, K, M) and LN229 (J, L, N) cells treated as in (G–H) and exposed to RSL3 (5 μM) under hypoxia for 24 h ∗*P* < 0.05,∗∗*P* < 0.01,∗∗∗*P* < 0.001,∗∗∗∗*P* < 0.0001. SCD1, stearoyl-CoA desaturase-1; MDA, malondialdehyde; LDH, lactate dehydrogenase.

To determine whether the regulation of SCD1 and FASN by SREBP1 occurs at the transcriptional level, we examined their mRNA expression by qPCR. Consistent with the protein data, hypoxia significantly induced the mRNA levels of SCD1 and FASN in shNC-transfected cells in both U251MG and LN229 cell lines. This induction was largely abrogated by SREBP1 knockdown under hypoxic conditions (Fig. 4C–F). In U251MG and LN229 cells, SCD1 mRNA levels were significantly higher in hypoxic control cells compared to normoxic controls (*P* < 0.001), and this increase was significantly attenuated by SREBP1 knockdown (*P* < 0.01) (Fig. 4C, D). Similarly, FASN mRNA expression was significantly upregulated by hypoxia in control cells (*P* < 0.01), and this effect was markedly reduced upon SREBP1 knockdown (Fig. 4E, F). These results demonstrate that hypoxia transcriptionally upregulates SCD1 and FASN expression through an SREBP1-dependent mechanism.

Having established that SREBP1 is required for hypoxia-induced SCD1 and FASN expression, we next investigated whether SREBP1-mediated ferroptosis resistance is dependent on its downstream target SCD1. To address this question, we performed rescue experiments by overexpressing SCD1 in SREBF1-knockdown glioma cells. U251MG and LN229 cells were transfected with shNC, shSREBF1, or cotransfected with shSREBF1 and an SCD1 overexpression plasmid (shSREBF1+OE SCD1), then cultured under hypoxic conditions. Western blot analysis confirmed efficient knockdown of SREBP1 and successful restoration of SCD1 protein levels in the rescue groups in both cell lines (Fig. 4G, H).

Functional assays revealed that SREBP1 knockdown significantly sensitized glioma cells to RSL3-induced ferroptosis under hypoxia. As shown in Fig. 4I, J, cell viability was markedly reduced in shSREBF1-transfected cells compared to shNC controls upon RSL3 treatment (*P* < 0.001). Consistently, lipid peroxidation, as measured by MDA levels, was significantly increased in SREBF1-knockdown cells (Fig. 4K-L, *P* < 0.01), and cytotoxicity, assessed by LDH release, was also significantly elevated (Fig. 4M-N, *P* < 0.01). Strikingly, overexpression of SCD1 in SREBF1-knockdown cells partially but significantly rescued these phenotypes. Compared to shSREBF1-transfected cells, the shSREBF1+OE SCD1 group exhibited significantly restored cell viability (Fig. 4I-J, *P* < 0.05), reduced lipid peroxidation (Fig. 4K, L, *P* < 0.05), and decreased LDH release (Fig. 4M, N, *P* < 0.05). These results demonstrate that SCD1 is a critical downstream effector of SREBP1 in mediating hypoxia-induced ferroptosis resistance.

Collectively, our findings establish that the SREBP1-SCD1 axis plays an essential role in protecting glioma cells from ferroptosis under hypoxic conditions. Hypoxia activates SREBP1, which transcriptionally upregulates SCD1 and FASN expression, leading to enhanced resistance to ferroptosis. The rescue experiments confirm that SCD1 is a key functional mediator of this protective effect.

### HIF-1α regulates SREBP1 activation and transcriptionally upregulates FASN and SCD1 under hypoxic conditions

To investigate the role of HIF-1α in regulating the SREBP1-SCD1/FASN axis under hypoxic conditions, we first confirmed the hypoxic response in our experimental system. U251MG and LN229 cells were cultured under normoxic or hypoxic conditions for 24 h. Western blot analysis revealed that hypoxia significantly stabilized HIF-1α protein levels in both cell lines (Fig. 5A), confirming the establishment of hypoxic conditions.

**Fig. 5:**
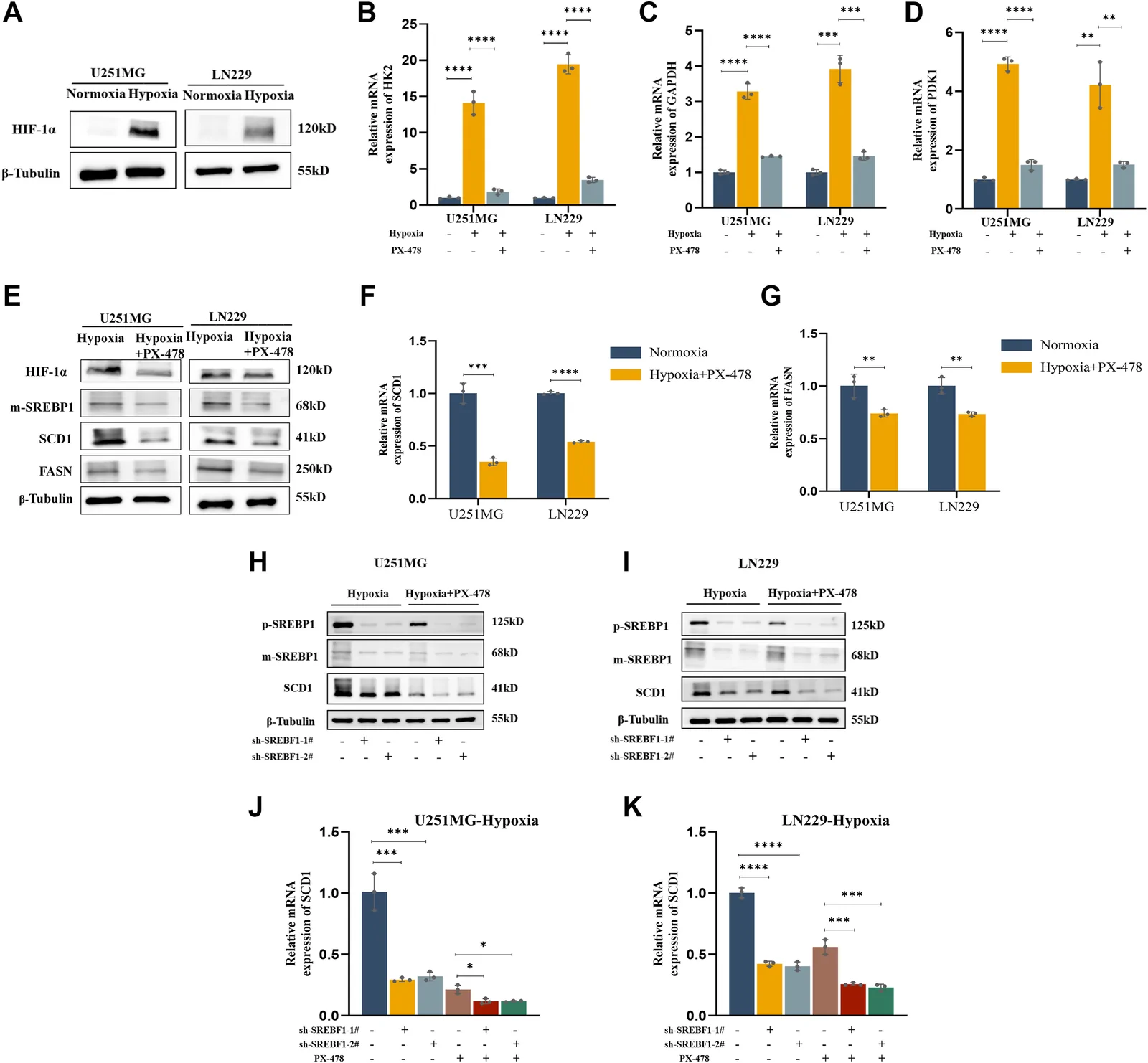
HIF-1α promotes SREBP1 nuclear translocation and transcriptional upregulation of FASN and SCD1. A: Western blot analysis of HIF-1α in U251MG and LN229 cells cultured under normoxia (N) or hypoxia (H) for 24 h. Protein quantitative analysis is shown in Supplementary file Figure 3A. B–D: qPCR analysis of HIF-1α target genes HK2 (B), GAPDH (C), and PDK1 (D) in U251MG and LN229 cells cultured under normoxia, hypoxia, or hypoxia with PX-478 (10 μM) for 24 h. E: Western blot analysis of HIF-1α, mature SREBP1 (m-SREBP1), SCD1, and FASN in U251MG and LN229 cells treated as in (B–D).Protein quantitative analysis is shown in Supplementary file Figure 3C–F. F and G: qPCR analysis of SCD1 (F) and FASN (G) mRNA levels in U251MG and LN229 cells treated as in (B–D). H and I: Western blot analysis of HIF-1α, m-SREBP1, and SCD1 in U251MG (H) and LN229 (I) cells transfected with shSREBF1 and cultured under hypoxia with or without PX-478 (10 μM) for 24 h.Protein quantitative analysis is shown in Supplementary file Figure 3G–I. J and K: qPCR analysis of SCD1 mRNA levels in U251MG (J) and LN229 (K) cells treated as in (H–I).∗*P* < 0.05,∗∗*P* < 0.01,∗∗∗*P* < 0.001,∗∗∗∗*P* < 0.0001. HIF, hypoxia-inducible factor; SCD1, stearoyl-CoA desaturase-1.

To further validate that hypoxia functionally activated HIF-1α transcriptional activity, we examined the mRNA expression of well-established HIF-1α downstream target genes, including HK2 (hexokinase 2), GAPDH (glyceraldehyde-3-phosphate dehydrogenase), and PDK1 (pyruvate dehydrogenase kinase 1). qPCR analysis showed that hypoxia significantly upregulated the mRNA levels of all three target genes in both U251MG and LN229 cells (Fig. 5B–D). Treatment with the HIF-1α-specific inhibitor PX-478 (10 μM) under hypoxic conditions effectively abrogated these increases, returning the mRNA levels of HK2, GAPDH, and PDK1 to near-normoxic levels (Fig. 5B–D). These results confirm that hypoxia activates HIF-1α transcriptional activity in our glioma cell models, and that PX-478 effectively inhibits HIF-1α function. Furthermore, we examined SREBF1 mRNA levels under hypoxic conditions by qPCR. Compared to normoxia, hypoxia significantly upregulated SREBF1 mRNA expression in both U251MG and LN229 cells (supplemental Fig. S3B).

Having validated the hypoxic model, we next examined the effect of HIF-1α inhibition on the SREBP1/SCD1/FASN axis. Consistent with our previous observations, hypoxia significantly increased the protein levels of HIF-1α, m-SREBP1, SCD1, and FASN in both U251MG and LN229 cells (Fig. 5E). Importantly, treatment with PX-478 under hypoxic conditions markedly attenuated the hypoxia-induced upregulation of m-SREBP1, SCD1, and FASN, while also reducing HIF-1α protein levels as expected (Fig. 5E). These results indicate that HIF-1α is required for the hypoxia-induced increase in m-SREBP1 protein levels and the subsequent upregulation of its downstream targets SCD1 and FASN.

To determine whether the regulation of SCD1 and FASN by HIF-1α occurs at the transcriptional level, we examined their mRNA expression following HIF-1α inhibition. qPCR analysis revealed that hypoxia significantly induced SCD1 and FASN mRNA levels in both U251MG and LN229 cells (Fig. 5F-G). Treatment with PX-478 under hypoxic conditions significantly inhibited the hypoxia-induced increase of SCD1 and FASN. (Fig. 5F-G). These results demonstrate that HIF-1α transcriptionally regulates SCD1 and FASN expression under hypoxic conditions.

To further dissect the regulatory relationship between HIF-1α and SREBP1, we performed combination experiments using both the HIF-1α inhibitor PX-478 and SREBP1 knockdown. U251MG and LN229 cells were transfected with shSREBF1 and cultured under hypoxia with or without PX-478 treatment. Western blot analysis showed that either SREBP1 knockdown or PX-478 treatment alone significantly reduced SCD1 protein levels under hypoxia (Fig. 5H, I). Notably, the combination of SREBP1 knockdown and PX-478 treatment resulted in a further reduction of SCD1 expression, suggesting that HIF-1α and SREBP1 may cooperatively regulate SCD1 expression through distinct but complementary mechanisms (Fig. 5H, I). Consistent with the protein data, qPCR analysis revealed that SCD1 mRNA levels were significantly reduced by either SREBP1 knockdown or PX-478 treatment alone, and the combination further decreased SCD1 expression in both cell lines (Fig. 5J, K).

Taken together, these results demonstrate that hypoxia upregulates SREBF1 mRNA and increases m-SREBP1 protein levels in a HIF-1α-dependent manner, while also transcriptionally activating SCD1 and FASN. These findings indicate that the regulation of the SREBP1-SCD1/FASN axis under hypoxia occurs at multiple levels, with HIF-1α playing a critical role in the activation of SREBP1 and the subsequent transcriptional upregulation of its downstream targets. However, the precise molecular mechanism by which HIF-1α regulates SREBP1—whether through direct transcriptional activation of SREBF1, modulation of SREBF cleavage-activating protein (SCAP) expression, or effects on SREBP1 cleavage and maturation—remains to be fully defined and warrants further investigation.

### Targeting HIF-1α reverses hypoxia-induced ferroptosis resistance and enhances RSL3 efficacy in vivo

To verify the function of HIF-1α in the regulation of ferroptosis in glioma cells under hypoxic conditions, we treated cells with PX-478 under hypoxia conditions. The results showed that inhibition of HIF-1α significantly enhanced RSL3-induced cell death (Fig. 6A), MDA level (Fig. 6B), and LDH release (Fig. 6C), and this effect could be partially reversed by DFO.

**Fig. 6:**
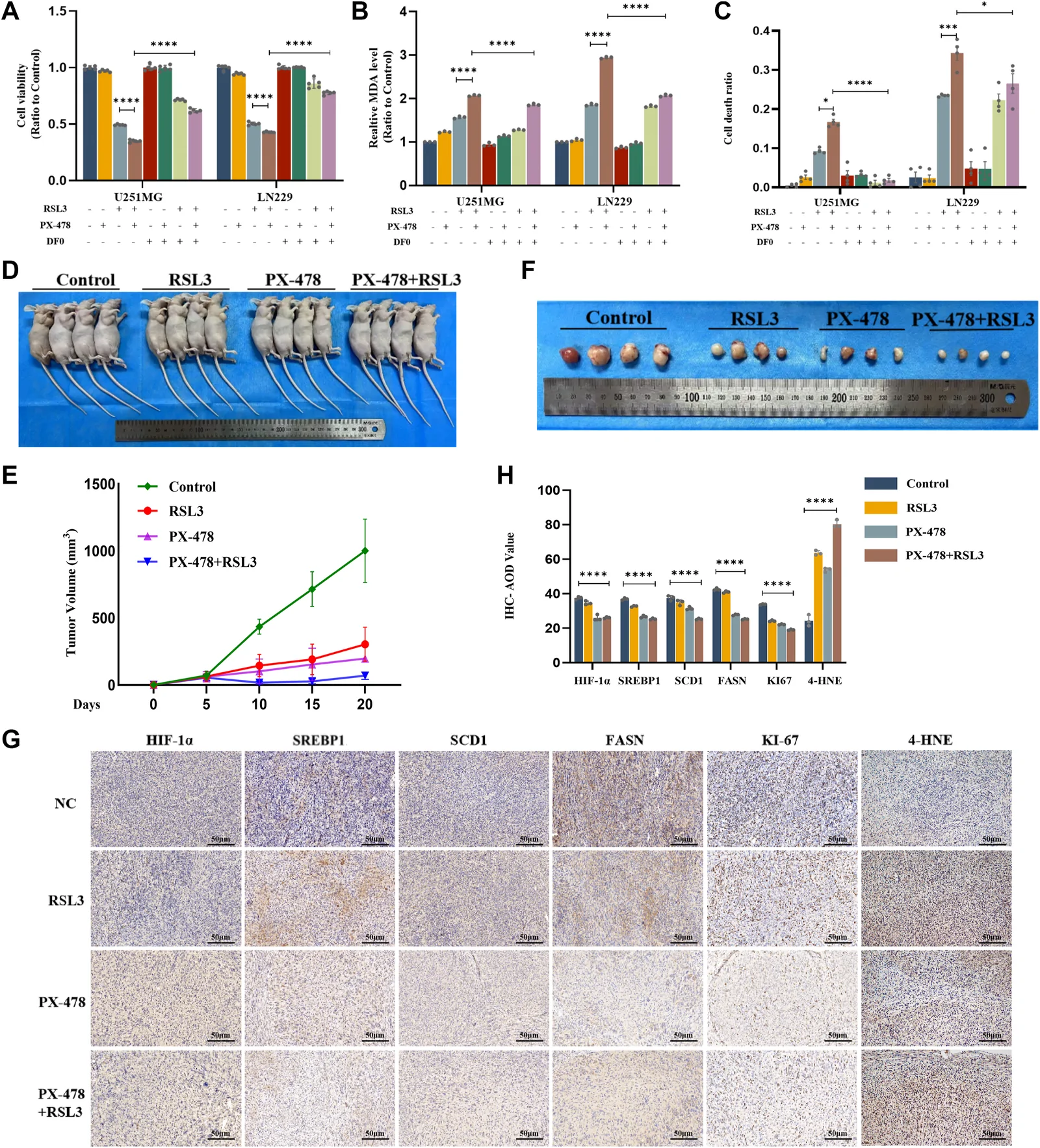
Targeting HIF-1α reverses hypoxia-induced ferroptosis resistance and enhances RSL3 efficacy in vivo. A–C: Cells were cultured under hypoxic conditions, and cells were treated with RSL3, PX-478 (10 μM), DFO or their combination for 24 h, and cell viability was detected by CCK-8, the degree of peroxidation by lipid peroxide (MDA), and the degree of cell death by LDH was detected. (D–F) Four groups of nude mice were injected intraperitoneally with normal saline, PX-478 (15 mg/kg), RSL3 (10 mg/kg), or PX-478 combined with RSL3 (15 mg/kg 10 mg/kg) every 3 days for 16 days. Images of nude mice and tumors from different groups. E: Changes in tumor volume in different groups. G, H: immunohistochemical staining was used to determine the expression and quantitative analysis of HIF-1α, SREBP1, SCD1, FASN, KI-67, and 4-HNE in different groups.∗*P* < 0.05,∗∗*P* < 0.01,∗∗∗*P* < 0.001,∗∗∗∗*P* < 0.0001. HIF, hypoxia-inducible factor; DFO, deferoxamine; LDH, lactate dehydrogenase; SCD1, stearoyl-CoA desaturase-1; 4-HNE, 4-hydroxynonenal.

*In vivo* experiments, we established a nude mouse xenograft model of U251MG glioma cells. After 5 days of tumor inoculation, the mice were randomly divided into four groups and given normal saline (control group), PX-478 (15 mg/kg), RSL3 (10 mg/kg), and PX-478 combined with RSL3. The experimental results showed that the tumor growth rate of the combination treatment group was significantly slower than that of the monotherapy group (Fig. 6D–F).

Immunohistochemical analysis of tumor tissues was performed to evaluate the expression of HIF-1α, SREBP1, FASN, SCD1, Ki-67, and 4-hydroxynonenal (4-HNE) across the four treatment groups (Fig. 6G, H). As expected, PX-478 treatment (alone or in combination with RSL3) significantly reduced the protein levels of HIF-1α and its downstream targets SREBP1, FASN, and SCD1 in tumor tissues, confirming effective on-target inhibition of the HIF-1α-SREBP1-FASN/SCD1 pathway in vivo. Furthermore, the Ki-67 positive rate in the combination treatment group was significantly lower than that in the monotherapy groups, indicating that the synergistic effect of PX-478 and RSL3 effectively inhibits glioma cell proliferation. To determine whether the enhanced antitumor efficacy was associated with ferroptosis induction in vivo, we performed immunostaining for 4-HNE, a well-established marker of lipid peroxidation. As shown in Fig. 6G, H, 4-HNE staining intensity was markedly elevated in the PX-478+RSL3 combination group compared to the control and monotherapy groups, providing direct evidence that HIF-1α inhibition sensitizes glioma tumors to RSL3-induced ferroptosis in vivo. Collectively, these results confirm that combining HIF-1α inhibition (PX-478) with ferroptosis induction (RSL3) exerts significant synergistic anti-tumor effects in glioma, with potential clinical translational value.

### The HIF-1α–SREBP1–FASN/SCD1 pathway was significantly activated in the tissues of glioma patients' tissues

Finally, we analyzed human glioma tissues by immunohistochemistry and found that the expression of HIF-1α, SREBP1, FASN, and SCD1 proteins was significantly increased in glioma samples compared to adjacent tissues (Fig. 7A–H). This result further supports the correlation of the mechanistic pathway proposed in this study in clinical samples, suggesting that the hypoxic microenvironment can promote lipid metabolism reprogramming and ferroptosis resistance through the HIF-1α–SREBP1–FASN/SCD1 axis, providing a clinical basis for the treatment strategy of this pathway.

**Fig. 7:**
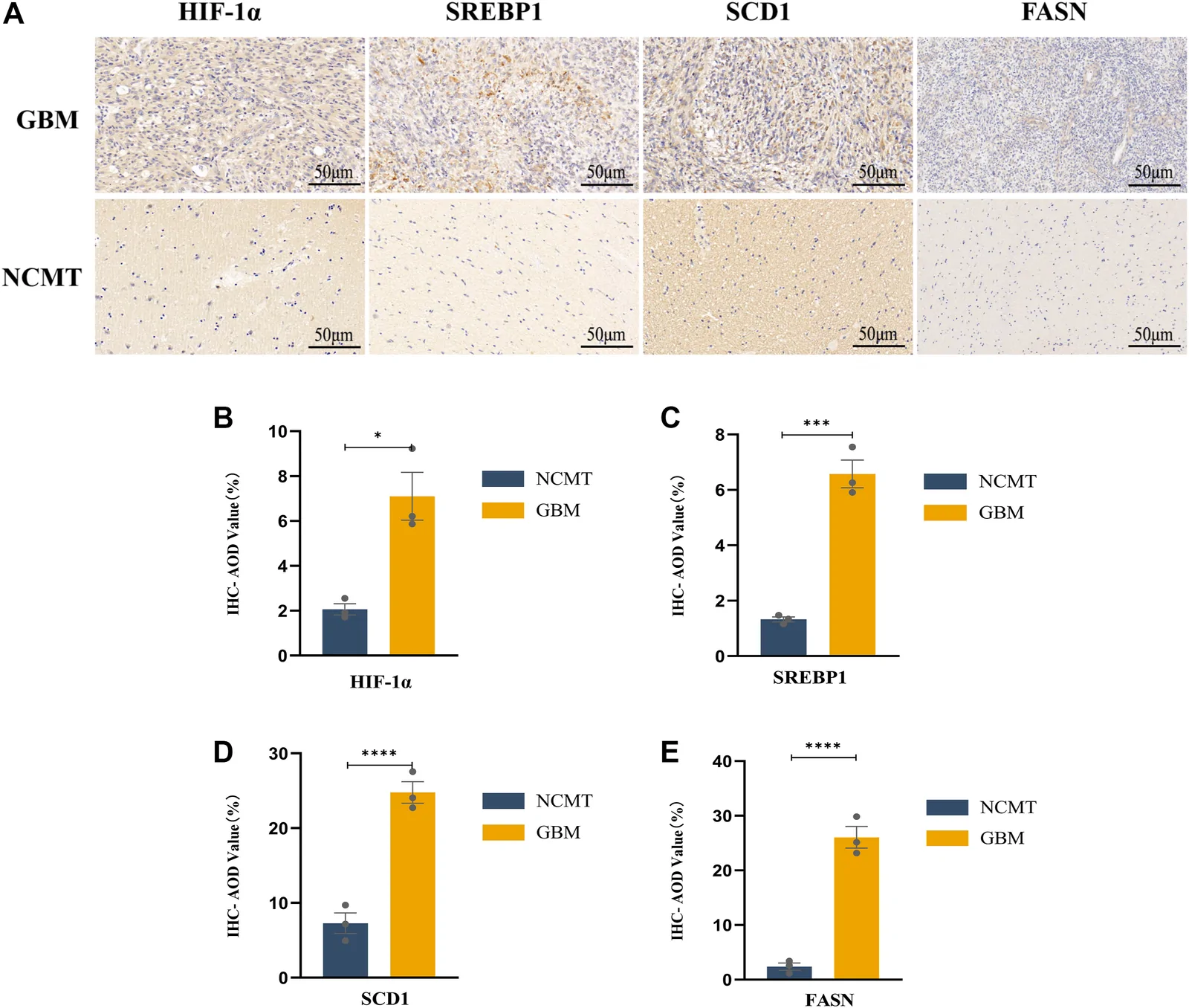
The HIF-1α–SREBP1–FASN/SCD1 pathway was significantly activated in glioma patients' tissues. (A–E) Immunohistochemical staining and quantitative analysis of HIF-1α, SREBP1, SCD1, and FASN were performed on tumor tissue and non-tumor tissue adjacent to cancer.∗*P* < 0.05,∗∗*P* < 0.01,∗∗∗*P* < 0.001,∗∗∗∗*P* < 0.0001. HIF, hypoxia-inducible factor; SCD1, stearoyl-CoA desaturase-1.

## Discussion

Glioma is the most common and aggressive primary tumor in the central nervous system, with a high degree of malignancy, poor prognosis, and limited effectiveness of existing treatment methods (25). Traditional chemotherapy drugs mainly kill tumor cells by inducing apoptosis, but gliomas often show resistance to apoptosis, leading to treatment failure. In recent years, ferroptosis, as an iron-dependent, lipid peroxidation-driven mode of cell death, has been thought to be able to bypass the apoptotic resistance mechanism, providing a new research direction for glioma treatment.

The occurrence of ferroptosis depends on the balance between intracellular iron metabolism, lipid peroxidation and antioxidant system. With the deepening of research, it has been found that tumor cells can also acquire resistance to ferroptosis through various mechanisms, such as upregulation of glutathione system and regulation of iron autophagy. Recent studies have shown that changes in lipid metabolism, particularly in membrane lipid composition, may also significantly affect cellular susceptibility to ferroptosis (26, 27, 28). Among them, increased MUFA synthesis mediated by SCD1 can inhibit lipid peroxidation by reducing the proportion of PUFAs, which in turn leads to ferroptosis resistance (26, 29, 30, 31, 32, 33, 34). Ferroptosis is driven by a chain reaction of lipid peroxidation targeting PUFAs within membrane phospholipids.In our lipidomics analysis, the cumulative increase in MUFA and SFA content across multiple phospholipid classes under hypoxia, while modest in individual species, collectively represents a biologically meaningful shift in membrane composition that contributes to the observed ferroptosis resistance. This is consistent with our functional data demonstrating that hypoxia confers significant protection against RSL3-induced ferroptosis in both glioma cell lines. During MUFA synthesis, key enzymes SCD1 and ACSL3 regulate cell membrane lipid composition by catalyzing fatty acid conversion and can indirectly regulate iron metabolism (35, 36). As a tumor type with significant reprogramming of lipid metabolism, the association between lipid metabolism changes and ferroptosis sensitivity needs to be further explored.

Hypoxia is a typical feature of the glioma microenvironment, and this persistent hypoxia not only promotes tumor angiogenesis and invasive metastasis, but also enhances the resistance of tumor cells to various stresses by driving metabolic reprogramming (37, 38, 39, 40, 41). Studies have suggested that hypoxia can significantly affect lipid metabolism (42, 43), but there is no clear study on whether it leads to ferroptosis resistance by regulating lipid metabolism in glioma. The purpose of this study was to investigate the regulatory mechanism of ferroptosis in glioma cells under hypoxia, with a special focus on the HIF-1α–SREBP1 signaling axis and its downstream lipid metabolizing enzymes FASN and SCD1 in inhibiting ferroptosis, in order to provide a new strategy for overcoming glioma treatment resistance. SREBP1 belongs to the SREBP transcription factor family and is a key factor in regulating cholesterol, fatty acids, and phospholipid synthesis genes (44, 45, 46). SREBP1 is typically present as an inactive precursor on the endoplasmic reticulum membrane and forms a complex with its chaperone protein SCAP. Studies have shown that SREBP1 expression is often located in hypoxic areas of tumors such as breast cancer, and HIF-1α silencing can reduce SREBP1 expression levels (47, 48).We acknowledge that the current in vivo evidence for ferroptosis induction is limited to 4-HNE immunohistochemistry, and we did not assess other markers such as MDA, lipid ROS, PTGS2, or perform transmission electron microscopy, primarily due to limited availability of archival tumor tissues after sectioning for multiple stainings. Nevertheless, the 4-HNE data, together with our robust in vitro evidence of increased MDA and LDH release upon HIF-1α inhibition, support the conclusion that the combination treatment enhances ferroptosis in vivo. Future studies using orthotopic models with more comprehensive ferroptosis marker panels will further validate these findings.

HIF-1α is a core transcription factor in the cellular response to hypoxia, regulating important adaptation processes including glycolysis and angiogenesis. This study found that the activation of HIF-1α significantly inhibited ferroptosis in glioma cells under hypoxic conditions, which may enhance cellular resistance to ferroptosis through lipid metabolism reprogramming. Further mechanistic studies showed that HIF-1α upregulated the expression of FASN and desaturase SCD1 by promoting the nuclear translocation of the transcription factor SREBP1, thereby increasing the proportion of MUFAs, reducing lipid peroxidation, and forming multiple defense mechanisms.Through genetic rescue experiments, we further demonstrated that SCD1 is a critical downstream effector of SREBP1 in mediating ferroptosis resistance. SREBP1 knockdown significantly sensitized glioma cells to RSL3-induced ferroptosis under hypoxia, and this effect was partially but significantly rescued by SCD1 overexpression. These results establish a causal relationship within the SREBP1-SCD1 axis and confirm that SCD1 is not merely a correlate but a functionally essential mediator of SREBP1-dependent ferroptosis protection. In addition, we observed that HIF-1α may regulate its expression by directly binding to the SCD1 promoter (49), and whether it can also directly regulate FASN expression by transcription remains to be investigated further. It is important to note that the current in vivo experiments, while providing proof-of-concept that PX-478-mediated HIF-1α inhibition can enhance ferroptosis sensitivity in tumors, were designed to broadly validate our in vitro mechanistic findings. The in vivo data confirm that targeting HIF-1α effectively reduces intratumoral FASN and SCD1 protein levels. However, these findings do not isolate the specific contribution of the SREBP1-FASN/SCD1 axis in vivo, as HIF-1α also regulates other pathways, such as SLC7A11. Therefore, a direct causal link between SREBP1/SCD1 activation and ferroptosis resistance in an in vivo glioma model remains to be established.

A critical point that warrants discussion is the oxygen dependency of SCD1 enzymatic activity. SCD1 is a di-iron enzyme that requires molecular oxygen as a cosubstrate, and it is well documented that severe hypoxia or anoxia can limit its activity (50). The hypoxic conditions used in this study (1% O_2_) represent mild to moderate hypoxia, where SCD1 likely retains partial but reduced catalytic activity. We therefore propose that the pronounced upregulation of SCD1 protein observed under hypoxia serves as a key compensatory mechanism: by increasing the number of enzyme molecules, cells can sustain sufficient MUFA synthesis to maintain membrane integrity and signaling functions essential for survival. To functionally test whether this hypoxia-induced MUFA pool, although modest in bulk lipidomics measurements, is sufficient to confer ferroptosis resistance, we performed ACSL3 knockdown experiments. ACSL3 catalyzes the conversion of free MUFAs to MUFA-CoA, an obligate step for their incorporation into membrane phospholipids, and its inhibition has been shown to prevent MUFA-mediated ferroptosis protection (22). Our results showed that ACSL3 knockdown sensitized glioma cells to RSL3-induced ferroptosis under both normoxia and hypoxia. More importantly, ACSL3 depletion largely reversed the ferroptosis resistance conferred by hypoxia, with cell death and lipid peroxidation levels approaching those observed under normoxia. These data provide direct functional evidence that the hypoxia-elevated MUFAs, once incorporated into membrane phospholipids, are functionally significant and capable of mediating a meaningful degree of ferroptosis resistance. This metabolic vulnerability explains why pharmacological inhibition of SCD1 with A939572 or genetic knockdown of SREBP1 produced such a dramatic reversal of ferroptosis resistance under hypoxia. Second, SCD1 may also contribute to ferroptosis resistance through functions beyond its canonical desaturase activity, such as modulating lipid droplet dynamics or membrane architecture. Third, the observed shift in overall fatty acid saturation—accumulated SFAs accompanied by modestly increased MUFAs—likely reflects a dynamic equilibrium in which partially inhibited SCD1 activity leads to SFA buildup, while compensatory upregulation of the enzyme maintains MUFA pools. Together, these findings support a model in which hypoxia imposes an oxygen-dependent constraint on SCD1 catalytic efficiency, forcing cells to upregulate the entire SREBP1-SCD1-ACSL3 axis to maintain protective MUFA levels in membrane phospholipids. This adaptation, while effective, creates a therapeutically exploitable metabolic vulnerability. Therefore, rather than simply being a consequence of a large-scale MUFA-driven alteration in membrane lipid composition, ferroptosis resistance under hypoxia likely stems from an integrated adaptive program centered on the HIF-1α-SREBP1-SCD1 axis.

Regarding the regulatory mechanism by which HIF-1α influences SREBP1, our data indicate that HIF-1α is crucial for SREBP1 activation and the subsequent upregulation of FASN and SCD1 under hypoxic conditions. In our experimental system, we observed that hypoxia not only increased m-SREBP1 protein levels but also upregulated SREBF1 mRNA expression, suggesting that hypoxia may regulate SREBP1 at both transcriptional and postranscriptional levels. However, we acknowledge that the precise mechanism by which HIF-1α regulates SREBP1 remains incompletely defined. Whether HIF-1α directly mediates the transcriptional upregulation of SREBF1, modulates the expression of its chaperone protein SCAP, or influences SREBP1 cleavage and maturation requires further investigation through approaches such as chromatin immunoprecipitation assays and nuclear-cytoplasmic fractionation experiments. It has been suggested that HIF-1α may transcriptionally regulate SREBP1 (48, 49), or that it could influence the SREBP1 pathway by modulating the expression of its chaperone protein, SCAP, which controls SREBP1 maturation and ER-to-Golgi transport (14). In addition, other studies have demonstrated that HIF-1α can directly promote SREBP1 protein expression (47), and multiple independent investigations have positioned activated HIF-1α upstream of SREBP1 in various cellular contexts (14, 51, 52). Our observation that HIF-1α inhibition with PX-478 dramatically reduces both SREBF1 mRNA and m-SREBP1 protein levels is consistent with these reports and suggests that HIF-1α may regulate SREBP1 through both transcriptional and posttranscriptional mechanisms. Future studies employing chromatin immunoprecipitation to assess HIF-1α binding to the SREBP1 or SCAP promoters, or examining SCAP expression levels under hypoxia with HIF-1α modulation, would be valuable to dissect the exact mechanism. Nevertheless, our findings clearly establish that HIF-1α is required for the hypoxia-induced activation of the SREBP1-FASN/SCD1 axis, providing a solid foundation for the mechanistic model proposed in this study.

In this study, lipidomics analysis also confirmed that the proportion of saturated and MUFAs in glioma cell membrane phospholipids increased and the proportion of PUFAs decreased after hypoxia treatment, which significantly inhibited the occurrence of ferroptosis. In terms of treatment strategy, the combination of HIF-1α inhibitor PX-478 and ferroptosis inducer RSL3 showed synergistic anti-tumor effects in vitro and in vivo experiments, suggesting that this combined strategy has clinical translation potential.

It is important to note that while PX-478 has been previously reported to synergize with ferroptosis inducers in glioma (24), the primary novelty of our study lies not in the use of PX-478 itself, but in elucidating the mechanistic link between the HIF-1α-SREBP1-FASN/SCD1 axis and hypoxia-induced ferroptosis resistance at the lipid metabolism level. Our in vivo experiment with PX-478 was designed as a proof-of-concept validation of our in vitro mechanistic findings, demonstrating that targeting HIF-1α can effectively reduce intratumoral FASN and SCD1 protein levels and enhance ferroptosis sensitivity in vivo. The choice of HIF-1α inhibitor over SREBP1 or SCD1 inhibitors was deliberate: hypoxia-activated HIF-1α confers ferroptosis resistance through multiple parallel pathways, including the SREBP1-FASN/SCD1 axis identified here and the SLC7A11-mediated antioxidant pathway reported previously (24). Therefore, HIF-1α inhibition would theoretically provide broader therapeutic benefits by simultaneously blocking multiple adaptive pathways. In addition, for intracranial glioma therapy, blood-brain barrier (BBB) penetrability is a critical consideration. Among available HIF-1α inhibitors, we selected PX-478 specifically because of its documented ability to cross the BBB, which likely explains its frequent use in glioma research.

We acknowledge several limitations regarding the in vivo experimental model used in this study. First, the subcutaneous xenograft model, while widely employed for initial efficacy evaluation, does not fully recapitulate the native brain microenvironment of glioma, including the BBB, glioma-associated parenchymal cells (such as microglia and astrocytes), and the unique immunosuppressive landscape of the central nervous system. Second, oxygen gradients and vascular perfusion characteristics differ substantially between subcutaneous and orthotopic sites, which may influence the hypoxic response and the efficacy of HIF-1α-targeted therapies. Third, the subcutaneous model does not account for the potential effects of the BBB on drug delivery, which is particularly relevant for PX-478. Therefore, while our subcutaneous xenograft data provide proof-of-concept evidence for the therapeutic potential of combining HIF-1α inhibition with ferroptosis induction, future studies employing orthotopic intracranial glioma models will be essential to validate these findings in a more physiologically relevant setting. In addition, while our in vivo validation is currently focused on targeting HIF-1α, the compelling in vitro data—particularly the SCD1 rescue experiments—suggest that directly inhibiting SREBP1 or SCD1 could represent a more specific strategy to overcome hypoxia-induced ferroptosis resistance. We acknowledge the lack of in vivo validation for these downstream targets as another limitation of the current study and an important direction for future research.

In conclusion, this study reveals a new mechanism by which gliomas reshape lipid metabolism through the HIF-1α-SREBP1/FASN/SCD1 signaling axis in the hypoxic microenvironment and thus resist ferroptosis, which provides a theoretical basis and experimental support for the development of combined therapies targeting the glioma microenvironment and cell death sensitivity. Future studies should focus on validating the efficacy of directly targeting SREBP1 or SCD1 in vivo and exploring the clinical translation of combining ferroptosis inducers with inhibitors of this adaptive pathway.

## Data availability

All data are available in the main text or supplementary materials.

## Supplemental data

This article contains supplemental data.

## Conflict of interest

The authors declare that they have no conflicts of interest with the contents of this article.
