# Supplementary material for: Hypoxia confers ferroptosis resistance in glioma cells via HIF-1α/SREBP1-mediated regulation of lipid metabolism
Source: J Lipid Res. 2026 Sep 1;67(10):101139. doi: 10.1016/j.jlr.2026.101139 (PMC13651567; doi:10.1016/j.jlr.2026.101139)
Supplement: Supplementary file 1 [file mmc1.docx]

**Supplementary file**

**1 shRNA , siRNA and RT-qPCR primer assay primer sequences**

shSREBP1#1 targeting sequence: GGAGCCATGGATTGCACTT

shSREBP1#2 targeting sequence: GCCATCGACTACATTCGCT

siHIF-1α#1 targeting sequence: AGAUGCGAACUCACAUUAUGTT

siHIF-1α#2 targeting sequence: GGAAAUGAGAGAAAUGCUUTT

siACSL3#1 targeting sequence: GCUGUGUAACAGUUGUGAAAU

siACSL3#2 targeting sequence: CCUGGAUGUGAUACUUUAGAU

β-Actin RT-qPCR Forward primer: TCGGAGTCAACGGATTTGGT

β-Actin RT-qPCR Reverse primer: TTCCCGTTCTCAGCCTTGAC

SREBF1 RT-qPCR Forward primer: GCCCCTGTAACGACCACTG

SREBF1 RT-qPCR Reverse primer: CAGCGAGTCTGCCTTGATG

SCD1 RT-qPCR Forward primer: AAACCTGGCTTGCTGATG

SCD1 RT-qPCR Reverse primer: GGGGGCTAATGTTCTTGTCA

FASN RT-qPCR Forward primer: TTCTACGGCTCCACGCTCTTCC

FASN RT-qPCR Reverse primer: GAAGAGTCTTCGTCAGCCAGGA

ACSL3 RT-qPCR Forward primer: CTTTCTCACGGATGCCGCATTG

ACSL3 RT-qPCR Reverse primer: CTGCTGCCATCAGTGTTGGTTTC

HIF-1α RT-qPCR Forward primer: CGCAAGTCCTCAAAGCACAGTTAC

HIF-1α RT-qPCR Reverse primer: ATTCATCAGTGGTGGCAGTGGTAG

Figure 1. (A-B)Validation of ACSL3 knockdown efficiency in U251MG (A) and LN229 (B) cells by qRT-PCR. (C-D) Cell viability assay in U251MG (C) and LN229 (D) cells under normoxia or hypoxia. Cells were treated with RSL3 (ferroptosis inducer), DFO (iron chelator), or si-ACSL3. (E-F) Relative MDA levels, a marker of lipid peroxidation, in U251MG (E) and LN229 (F) cells under normoxia or hypoxia with indicated treatments.(G-H) Cell death ratio in U251MG (G) and LN229 (H) cells under normoxia or hypoxia following RSL3, DFO, or si-ACSL3 treatment.* *P* < 0.05，** *P* < 0.01，*** *P* < 0.001，**** *P* < 0.0001.

Figure 2. (A-B) Quantitative analysis of protein levels of FASN and SCD1 by Western blot by incubating cells under normoxic and hypoxic conditions for 24 hours. (C-D) cells were transfected with sh-SREBF1 and silencing efficiency was verified using RT-qPCR. (E-H) cells were transfected with sh-SREBF1 and cultured under normoxic and hypoxic conditions for 24 hours, and the levels of m-SREBP1, p-SREBP1, SCD1 and FASN proteins were quantitatively analyzed by Western blot.* *P* < 0.05，** *P* < 0.01，*** *P* < 0.001，**** *P* < 0.0001.

Figure 3. (A) Quantitative analysis of protein levels of HIF-1α by Western blot by incubating cells under normoxic and hypoxic conditions for 24 hours. (B) Cells were cultured under normoxic and hypoxic conditions for 24 hours, and the mRNA levels of SREBF1 were detected by quantitative real-time PCR (qPCR). (C) cells were cultured under hypoxic conditions and treated with PX-478 (10 μM) for 24 h for quantitative analysis of HIF-1α protein levels by Western blot. (D-F) cells were cultured under hypoxic conditions and treated with PX-478 (10 μM) for 24 h for quantitative analysis of m-SREBF1, SCD1, and FASN protein levels by Western blot.(G-I) cells were transfected with sh-SREBF1 and cultured under hypoxic conditions, treated with PX-478 (10 μM) for 24 hours, and quantified by Western blot for the quantification of p-SREBP1, m-SREBP1, SCD1 and FASN protein levels.* *P* < 0.05，** *P* < 0.01，*** *P* < 0.001，**** *P* < 0.0001.
